# BitTorious volunteer: server-side extensions for centrally-managed volunteer storage in BitTorrent swarms

**DOI:** 10.1186/s12859-015-0779-6

**Published:** 2015-11-04

**Authors:** Preston V. Lee, Valentin Dinu

**Affiliations:** Department of Biomedical Informatics, Arizona State University, 13212 East Shea Boulevard, Scottsdale, AZ 85259 USA

**Keywords:** Data transfer, Bioinformatics, Big data, Software, Open source, BitTorrent, BitTorious, Citizen scientist

## Abstract

**Background:**

Our publication of the BitTorious portal [1] demonstrated the ability to create a privatized distributed data warehouse of sufficient magnitude for real-world bioinformatics studies using minimal changes to the standard BitTorrent tracker protocol. In this second phase, we release a new server-side specification to accept anonymous philantropic storage donations by the general public, wherein a small portion of each user’s local disk may be used for archival of scientific data. We have implementated the server-side announcement and control portions of this BitTorrent extension into v3.0.0 of the BitTorious portal, upon which compatible clients may be built.

**Results:**

Automated test cases for the BitTorious Volunteer extensions have been added to the portal’s v3.0.0 release, supporting validation of the “peer affinity” concept and announcement protocol introduced by this specification. Additionally, a separate reference implementation of affinity calculation has been provided in C++ for informaticians wishing to integrate into *libtorrent*-based projects.

**Conclusions:**

The BitTorrent “affinity” extensions as provided in the BitTorious portal reference implementation allow data publishers to crowdsource the extreme storage prerequisites for research in “big data” fields. With sufficient awareness and adoption of BitTorious Volunteer-based clients by the general public, the BitTorious portal may be able to provide peta-scale storage resources to the scientific community at relatively insignificant financial cost.

## Background

Individual .torrent files hold compartmentalized metadata treating the torrent payload as a sequence of equally-sized “pieces”, calculated at time of torrent creation. Hashes for each piece are included in the .torrent metadata file, which allow clients to validate the correctness and completeness of transferred data.

Under traditional BitTorrent use, users run individual BitTorrent clients with the intention of downloading the entirety of a given torrent’s payload to local disk, often seeding those data to other peers. That is, *every* piece listed in the .torrent file will be download and assembled by each client using the long-standardized and well understood BitTorrent specification. Our BitTorious v2.x.x portal release [1] supports these use cases while introducing strict *publisher* and *subscriber* role semantics as an access control layer not present in most BitTorrent networks. (The reader is encouraged to review this work [1] for a deeper explanation of core BitTorious concepts, user role types and their relation to standard BitTorrent.)

The needs of a client in a volunteer storage grid are fundamentally different from most studies of peer behavior [2–4]. We cannot assume the user desires to provide storage for the *entirety* of a single torrent, even if they have sufficient disk space as well as available bandwidth. For example, a user donating storage towards a typical WGS project will almost certainly *not* want to contribute more than a mere fraction of the disk space required for even a single patient study. For this reason alone, existing BitTorrent portals oriented toward distribution of scientific data cannot reasonably expect participation from citizen scientists, as there are no built-in mechanisms for centrally access controlled, partial replication.

Additionally, piece selection mechanisms vary between client implementations and target audience. A client oriented towards distribution of video files may, for example, deviate from standard piece selection by favoring pieces at the beginning of each file, thus supporting real-time video streaming prior to download completion. Any such piece and torrent prioritization algorithms active within a given BitTorrent client are or little, if any, use to a BitTorious client, and are likely to impair the resiliency of the BitTorious network by disproportionately over-replicating certain pieces since BitTorrent client developers tend to assume that the *user* chooses the torrents to join, not the tracker.

BitTorious v3.0.0 addresses these core usage differences using the publisher/subscriber security model unchanged from 2.x.x releases.

## Implementation

Building upon the existing role-based, per-user-per-feed security model supported by the BitTorious portal [1], we introduce several new concepts and capabilities in order to support controlled, partial replication.

### Publisher feed configuration

**Fig. 1:**
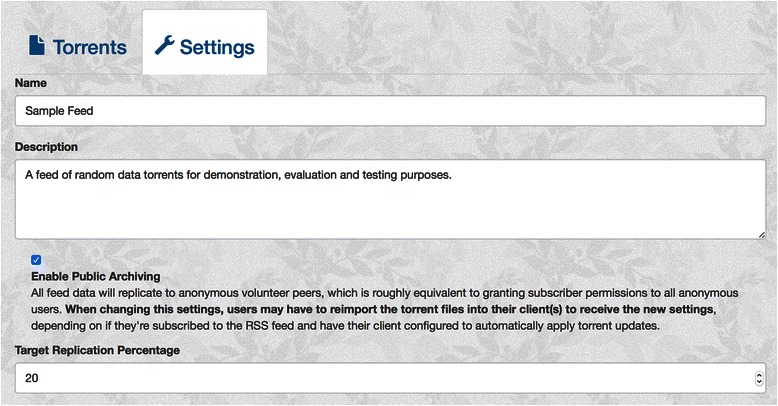
Feed-level volunteer configuration. The BitTorious portal has been extended to support several new feed-level configuration parameters: an “enabling public archiving” flag and “target replication percentage”

The BitTorious portal has been extended to support a number of new configuration options at the *feed* level that are used by the built - in BitTorrent tracker to assign piece prioritization across peers (See Fig. 1).

1. **Enable Public Archiving** (default: disabled) - When enabled, all volunteer clients will be able to donate storage to the feed. When enabled, the “private” flag will **not** be set for each torrent, which is automatic in BitTorrious v2.x.x.
2. **Target Replication Percentage** (default: 20 %, min: 1 %, max: 100 %) – Approximate percentage of pieces that a volunteer should replicate for a given torrent. The effective piece count will always be rounded up, and applies to all torrents published to the feed. A low percentage increases the capacity of the network at the expense of limited piece replication, while a higher value favors high piece replication to maximize availability at the expense of capacity.

### Piece affinity

BitTorious v3.0.0 introduces a concept we call “piece affinity”, which must be understood and calculated by the portal as well as fully compatible peers. The *affinity* of a peer declares the pieces it intends to replicate and seed. Affinity values are calculated via a simple, pre-defined function based on publisher-defined feed configuration as well as peer ID, thus enabling coordinated partial replication across the entire network and a limited degree of peer-peer affinity enforcement as every peer is able to calculate the affiny of every other peer without querying the portal.

All BitTorrent swarms require that all *N* pieces of a torrent must be of the same length, in bytes. (BitTorious recommends a piece size of exactly 4MiB, i.e., 2 ^ 22 bytes). Unlike standard BitTorrent, volunteer clients should *only* download pieces to which they are affine, though this cannot be enforced by the server since P2P transfers do not, by definition, route through the server. Also, strict server enforcement would likely introduce incompatibilities with normal, non-BitTorious-aware BitTorrent clients.

For a volunteer client to be compatible with the BitTorious network in a well-behaved manner, it must:

1. Inform the tracker of configuration parameters when announcing.
2. Obey a user-configurable local storage limit.
3. Delete non-affine pieces if *and only if* disk space is required for affine pieces or requested by the user.
4. Keep all portal/tracker interactions over HTTPS.
5. Delete all pieces for a given torrent when the time since last successful announce passes a pre-set time period.
6. Only send a given peers pieces to which it’s affine, regardless of what peers request.

Outside of these core semantic differences, a volunteer client uses the existing BitTorrent peer wire protocol.

### Volunteer announce parameters

At announcement time, volunteer peers must provide support for additional announce request parameters. By requiring these self-reported settings to the portal during the regular, periodic peer “announce” request process, the tracker may intelligently rebalance the network, if desired, as well as provide reporting capabilities to the feed publisher. The portal is thus also able to differentiate between BitTorious-enabled clients and normal BitTorrent clients.

**Table:** 

Key	Example	Type	Required?
volunteer [enabled]	1	Only a value of “1” will be recognized by the tracker as a volunteer announcement.	Yes
volunteer [disk_maximum_bytes]	8589934592	The hard storage limit, in bytes, as set by the user.	Yes
volunteer [disk_used_bytes]	1234567890	The amount of storage, in bytes, used by the client on the user’s machine.	Yes

To facilitate the tracking process, the BitTorious tracker observes a “volunteer = 1” option. Note that the semantic of the “left” value (the number of missing bytes across the entire torrent) submitted on peer announcement is unchanged, even if the client never intends on replicating remaining pieces. That is, it is perfectly normal for a volunteer client’s “left” to never reach zero, and a value below a calculatable threshold to be a symptom of a misbehaving client.

Unlike standard BitTorrent, BitTorious also defines a Target Replication Percentage, P, of the total number of pieces, N, sent to each client as part of the “announce” response. The value being expressed is an integer between 1 (inclusive) and 100 (inclusive), which is the maximum percentage of pieces, rounded up to the nearest integer, that all volunteer clients should keep locally replicated. The value is simply ignored by “normal” BitTorrent publisher and subscriber clients unaware of the volunteer extensions. The total number of pieces a client must download per torrent, M, is thus the same for every volunteer participating in the torrent, and is always rounded up to the nearest integer value.

$$ \mathrm{M} = \mathrm{ceiling}\left(\mathrm{N}\ *\ \mathrm{P}\ /\ 100.0\right) $$

All M pieces download by a given client must be limited to a contiguous region of piece numbers starting at an “Affinity Offset”, A, defined by each client as:

$$ \mathrm{A}=\mathrm{BASE}10\left(\mathrm{S}\mathrm{H}\mathrm{A}256\left(\mathrm{peer}\_\mathrm{id}\right)\right)\%\left(\mathrm{N}\hbox{-} 1\right) $$

Since the Peer ID is known to both peer and tracker, A does not technically need to be explicitly exchanged. Both M and A are returned as part of the announce response, however, as respective “volunteer[affinity_length]” and “volunteer[affinity_offset]” integers to allow clients to join without needing to first fetch feed configuration data, as well as for validation purposes. The last piece in the offset to be downloaded, L, is defined as follows, but is not necessarily reflective of the order in which pieces will be acquired:

$$ \mathrm{L} = \left(\mathrm{A} + \mathrm{M}\ \hbox{-}\ 1\right) $$

A client should attempt to locally replicate a given piece number, X, if and only if the following function evaluates to true:

should_replicate (X) : = (X > = A & & X < = L) || (N < = L & & X < = L ‐ N)

**Fig. 2:**
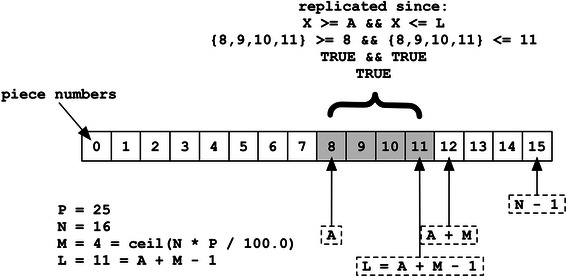
Affinity common case. A volunteer client’s view of a torrent with 16 pieces published to a feed with configuration computing to 4 torrent pieces per client, where the client’s affinity offset is 8. The range of affine pieces is contiguous

**Fig. 3:**
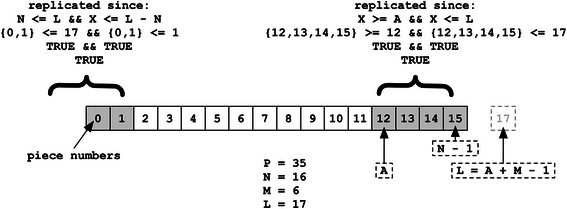
Affinity “wrap-around” case. A volunteer client’s view of a torrent with a “wrap-around” affinity. In this example N is still 16, but P has been raised to 35 % and A happens to be 12. Since L is greater than or equal to N, the first two pieces will also be included in the clients acquisition list

**Fig. 4:**
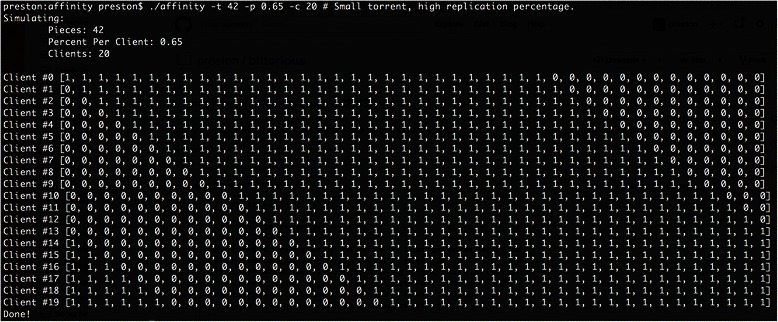
Command-line affinity calculation. The C/C++ command-line utility allows you to quickly visualize how piece affinity will be calculated prior to configuring a feed

The first half of the expression handles common cases where every block has a sequential piece number (see Fig. 2). This fails, however, when the pieces per torrent, M, would require a piece “past” the end of the torrent (see Figs. 3 and 4). In this scenario, the latter half of the expression simply allows the piece range to “wrap around” to the beginning of the torrent data at piece offset #0. These extensions in addition to corresponding GUI changes encapsulate the portal changes made for BitTorious v3.0.0 tracker and portal GUI.

**Fig. 5:**
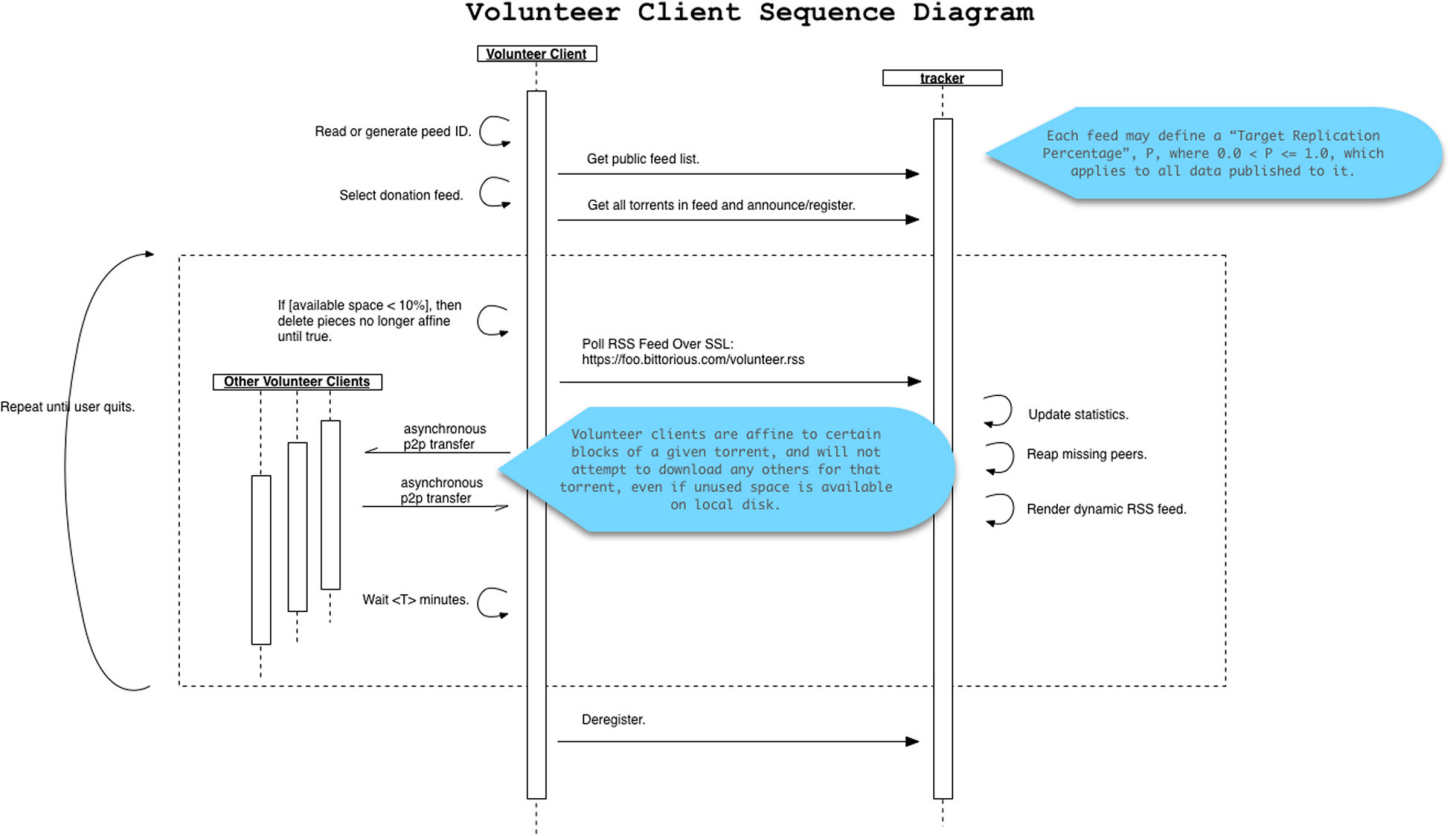
Volunteer client sequence diagram. The announcement and piece acquisition process used by a compatible volunteer client

The portal *may*, in future releases, provide a piece prioritization component by returning a dynamic piece acquistion priority *order* as part of the volunteer announcement response, but expects no specific piece prioritization in v3.x.x releases. Leaving piece order in the hands of client implementations allows for use of existing piece selection and peer choke/unchoke logic inherent to every BitTorrent client, given those pieces are within the assigned affiny range (see Fig. 5). In such event, the tracker will return an ordered array within the root level of the dictionary keyed by “piece_priority” when the client “volunteer” bit is present in the announcement as defined above, with higher priority pieces listed first. The client should obey this request when observed, but is not strictly required to do so since implementation inherently requires further modification of peer choking/unchocking determination.

## Results

We demonstrate affinity calculation with *two* reference implementations, both distributed as part of the portal source code. The first is written in Ruby and built into the portal’s peer registration logic. Automated regression test cases are provided to validate correct volunteer announcement extension behavior as well as server-side affinity calculation.

The second is a standalone command-line application written in C++ to quickly test how affinity will be calculated by the server (and compatible clients) for any valid feed configuration that may be easily integrated into existing C/C++ BitTorrent clients, such as those based on the popular “libtorrent” C++ library. Both implementations are released under unencumbered Open Source-compatible licenses.

## Discussion

Notable existing computing platforms such as BOINC have long proved the viability of using crowd sourced resources in real-world research, but have evolved with a strong skew towards *time*-heavy computing: algorithms that divide a problem into small distributed computing tasks later assembled to conquer the whole. Users of BOINC must abide by BOINC-specific API and prototcol mechanisms [5, 6] only well known within scientific communities. BitTorious is equal and opposite in this regard, providing centralized control of a storage “grid”, but designed foremost to address *space*-heavy storage of scientific data, not CPU cycles. BitTorious maintains direct compatibility with the most popular, widely known and best understood algorithms – BitTorrent – and leaves the existing core “peer wire protocol” unchanged, allowing voluminous amounts of research from other BitTorrent-based systems to be adapted far easier [7].

Developers implementing compatible clients are strongly encouraged to allow user control of when the client runs, where on local disk to put the data, a hard upper donation limit, GUI mechanism for selecting the feed(s) to which the resources will be donated, and automatic disabling of transfers while on “metered” Internet connections. Additionally, support for uPnP of NAT devices is very strongly recommended to facilitate inbound network connections without manual port forwarding configuration [8]. Failure to address the reality of home firewalls will significantly limit the overall network performance of a BitTorious-compatible client.

## Conclusions

In addition to clear functional potential, we observe our place in human evolution as marked by the advent of the *citizen scientist*. Legal, philosophical and ethical battles regarding regulation of “medical devices” (as they pertain to bioinformatics specifically) and digital governance (of BitTorrent networks in general [9, 10]) in American government are far from over, but the expectation of direct engagement with scientific communities has been set. Philanthropically, users expect voluntary contributions to be *direct*, *online*, and with *clear accountability* of how contributions are allocated. BitTorious’ v3.0.0 extensions fall in line with these cultural expectations by enabling the public to individually donate local storage resources to specific projects of their choosing via standards-compliant clients provided by the research community. Such a simple, direct and individually actionable mechanism for volunteer storage does not exist in practice.

The value of networks such as BitTorious, while not constrained by any fundamental limits of technological possibility, is limited by the magnitude of its user base. Any such effort to build a significantly sized storage network based on BitTorious must be met with a proportional effort in volunteer recruitment. Notwithstanding, introduction of a simple piece affinity mechanism as presented here is paramount to respecting the generous but limited contributions of volunteer peers. Without such a partial replication function, any general-purpose P2P technology is unlikely to be met with success in big data fields where payload size often exceeds locally available storage resources by an order of magnitude or more.

The adaptation of BitTorrent algorithms and protocols to public volunteer computing is important in its own right, but more importantly, has far-reaching potential to change the fundamental *economics* of 21st-century science. To the scientific community, the potential cost savings resultant of using a large volunteer network of partial-replication peers is most dramatic when considered at scale. For example, a theoretical personalized medicine trial syndicating a total of 1 PB of data into a single feed across 1000 torrents would average 1 TB of individual study data per torrent. At a minimum 1 % default Target Replication Percentage and volunteer maximum donation mode of 20GB, peers would hit their local 20GB device limit before reaching the 1 %-per-torrent (10GB) target threshold. To achieve 4x redundancy of each byte in the entire syndicated feed – that is, 4 distributed copies exclusive of the original seed copy – approximately 2 million volunteer devices are needed on the network. While lofty, this is significantly less than the 3 million active volunteer compute devices on the World Community Grid [11] network. Using 1 PB of Amazon S3 reduced-redundancy pricing as a baseline, we estimate the annual storage savings of this network alone to be in excess of $300,000 (USD) annually.

Openly available P2P technologies have disrupted the entertainment industry enough to create entirely new means of distributing artistic works, and BitTorious aims to do the same for scientific data. BitTorious decouples distributed storage from any singular domain, geographic, institutional or national interest, and if run at an adoption scale similar to World Community Grid would provide peta-scale distribution and archival resources to scientists in every domain worldwide.

## Availability and requirements

The BitTorious portal software may be downloaded and deployed via the public source code repository at https://github.com/preston/bittorious. Non-administrative, subscriber-only evaluation may be performed by requesting an account from the demo site, below, in conjunction with the free uTorrent client software.

- Project name: BitTorious
- Project home page:
  - **○** Source code (portal): https://github.com/preston/bittorious
  - **○** Demo: https://try.bittorious.com
  - **○** Tutorial: https://try.bittorious.com/getting_started
- **Operating system(s):** Both portal and clients are platform independent.
- **Programming language:** Ruby on Rails, AngularJS, Bootstrap, PostgreSQL.
- **Other requirements:** Ruby 2.2.1 or higher.
- License: MIT
- Any restrictions to use by non-academics: None

## References

[1] Lee PV, Dinu V (2014). BitTorious: global controlled genomics data publication, research and archiving via BitTorrent extensions. BMC Bioinformatics.

[2] Kash IA, Lai JK, Zhang H, Zohar A (2012). Economics of BitTorrent Communities.

[3] Adamsky F, Khayam SA, Jager R, Rajarajan M. Who Is Going to Be the Next BitTorrent Peer Idol? 2014 12th IEEE International Conference on Embedded and Ubiquitous Computing (EUC). 2014:293–298. doi:10.1109/EUC.2014.50.

[4] Cai QC, Lo KT (2014). An analysis of user behavior in a private BitTorrent community. Int J Commun Syst.

[5] Anderson DP. Boinc: A system for public-resource computing and storage. GRID '04 Proceedings of the 5th IEEE/ACM International Workshop on Grid Computing. 2004;4-10. doi:10.1109/GRID.2004.14.

[6] Elwaer A, Harrison A, Kelley I, Taylor I. Attic: A Case Study for Distributing Data in BOINC Projects. Distributed Processing, Workshops and Phd Forum (IPDPSW). 2011:1863–1870. doi:10.1109/IPDPS.2011.348.

[7] Leon X, Chaabouni R, Sanchez-Artigas M, Garcia-Lopez P (2014). Smart Cloud Seeding for BitTorrent in Datacenters. IEEE Internet Computing.

[8] Liu Y, Chang L, Pan J (2014). On the performance and fairness of BitTorrent-like data swarming systems with NAT devices. Comput Netw.

[9] Druckerman JA. The Uncertifiable Swarm: Why Defendant Class Actions and Mass BitTorrent Copyright Litigation Don't Mix. NYL Sch L Rev. Hein Online. 2013;931.

[10] Foreman VS. Problems with BitTorrent Litigation in the United states: Personal Jurisdiction, Joinder, Evidentiary Issues, and Why the Dutch Have a Better System. Wash U Global Stud L Rev. Hein Online. 2014;127.

[11] World Community Grid: Global Statistics. Downloaded September 3, 2015 from http://www.worldcommunitygrid.org/stat/viewGlobal.do; 2015.

